# Impaired coenzyme A synthesis in fission yeast causes defective mitosis, quiescence-exit failure, histone hypoacetylation and fragile DNA

**DOI:** 10.1098/rsob.120117

**Published:** 2012-09

**Authors:** Takahiro Nakamura, Tomáš Pluskal, Yukinobu Nakaseko, Mitsuhiro Yanagida

**Affiliations:** 1 Okinawa Institute of Science and Technology Graduate University, Tancha 1919-1, Onna, Okinawa 904-0495, Japan; 2 Graduate School of Biostudies, Kyoto University, Sakyo-ku, Kyoto 606-8501, Japan

**Keywords:** acetyl-CoA, pantothenate, phosphopantothenoylcysteine synthetase, lipid droplet, centromere/kinetochore

## Abstract

Biosynthesis of coenzyme A (CoA) requires a five-step process using pantothenate and cysteine in the fission yeast *Schizosaccharomyces pombe*. CoA contains a thiol (SH) group, which reacts with carboxylic acid to form thioesters, giving rise to acyl-activated CoAs such as acetyl-CoA. Acetyl-CoA is essential for energy metabolism and protein acetylation, and, in higher eukaryotes, for the production of neurotransmitters. We isolated a novel *S. pombe* temperature-sensitive strain *ppc1-537* mutated in the catalytic region of phosphopantothenoylcysteine synthetase (designated Ppc1), which is essential for CoA synthesis. The mutant becomes auxotrophic to pantothenate at permissive temperature, displaying greatly decreased levels of CoA, acetyl-CoA and histone acetylation. Moreover, *ppc1-537* mutant cells failed to restore proliferation from quiescence. Ppc1 is thus the product of a super-housekeeping gene. The *ppc1-537* mutant showed combined synthetic lethal defects with five of six histone deacetylase mutants, whereas *sir2* deletion exceptionally rescued the *ppc1-537* phenotype. In synchronous cultures, *ppc1-537* cells can proceed to the S phase, but lose viability during mitosis failing in sister centromere/kinetochore segregation and nuclear division. Additionally, double-strand break repair is defective in the *ppc1-537* mutant, producing fragile broken DNA, probably owing to diminished histone acetylation. The CoA-supported metabolism thus controls the state of chromosome DNA.

## 2. Introduction

Coenzyme A (CoA) is a ubiquitous, essential cofactor that plays a central role in the metabolism of carboxylic acids and lipids [1]. About 4 per cent of all known enzymes use CoA as an obligate cofactor. Therefore, CoA is involved in over 100 different reactions of intermediary metabolism [2,3]. CoA was discovered through a study on amino group acetylation of small molecules [4,5]. CoA was subsequently shown to be composed of adenosine 5′-phosphate, pantothenate and a sulphhydryl moiety. Acylation (thioesterification) of CoA at the sulphhydryl group by various carboxylic acids results in the production of many important acylated CoAs, including acetyl-CoA. Given that acetyl-CoA acts as the donor of acetyl group to numerous proteins, including histones by protein acetyltransferases, and protein acetylation is one of the principal post-translational protein modifications [6], the biosynthesis of CoA may be important for a number of cell regulations, including nutrient metabolism. In higher organisms, choline acetyltransferase produces the neurotransmitter acetylcholine by combining acetyl-CoA and choline.

**Figure 1.**
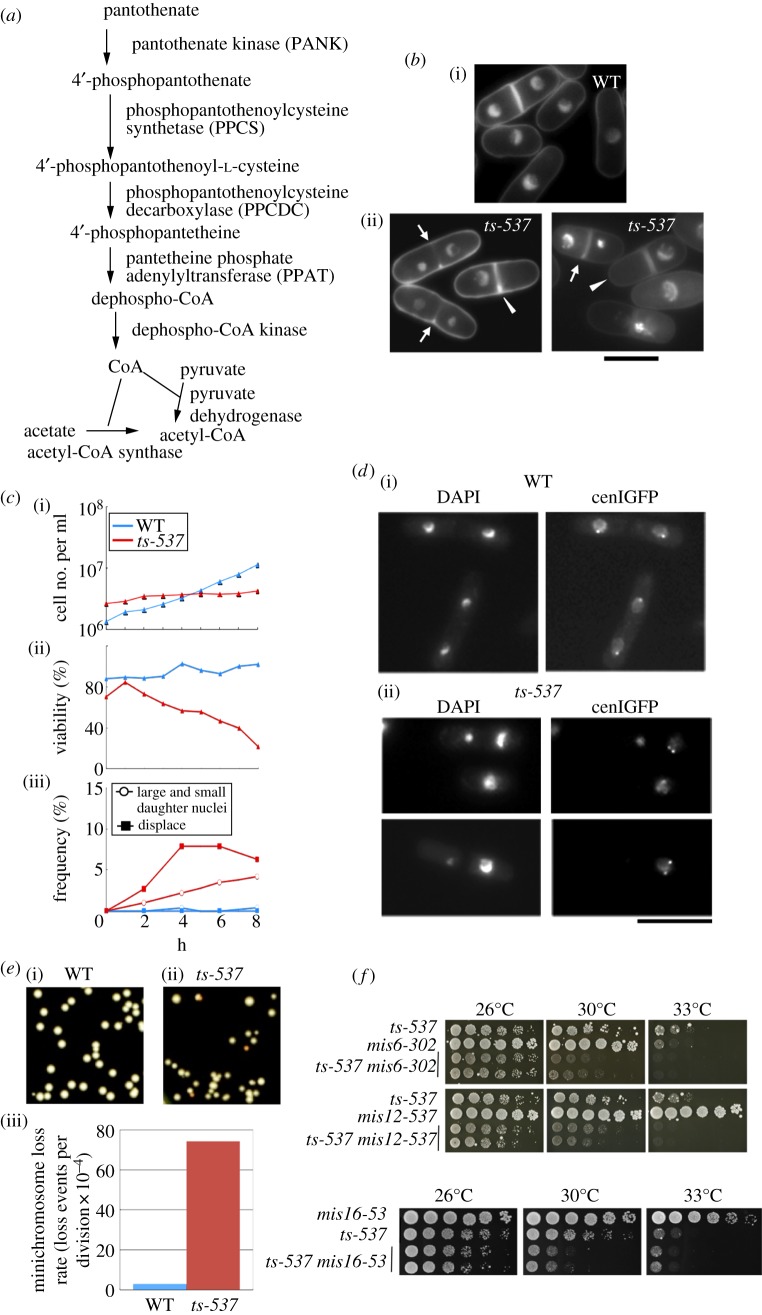
*ts-537* strain is defective in the accurate chromosome segregation. (*a*) The biosynthetic pathway of CoA from pantothenate. (*b*) Light micrographs of WT and ts mutant *ts-537*, which were cultured at 36°C for 4 h. Cells were fixed and stained with DAPI. Scale bar, 10 μm. The arrows and arrowheads indicate cells with the defective phenotypes of unequal segregation and displaced nucleus, respectively. (*c*) The WT and mutant *ts-537* cells exponentially grown at 26°C in the complete YPD medium were transferred to 36°C for 0–8 h. The cell number and cell viability percentage were scored at 1-h intervals under microscope and by plating. Aliquots of cells were fixed and stained with DAPI, and the frequency of aberrant mitosis was scored under the microscope. See text. (*d*) WT and *ts-537* strains chromosomally integrated with GFP-tagged Lac repressor that was bound to the peri-centromeric region of chromosome I (§5) were cultured at 36°C for 4 h, then fixed with methanol. The peri-centromeric GFP signals are shown together with DAPI staining. Scale bar, 10 μm. (*e*) WT and *ts-537* strains carrying an artificial minichromosome Ch10-CN2 were cultured in YPD medium at 26°C for 10 generations and plated on the complete medium. The colonies with high frequencies of minichromosome loss showed red–orange colour. The frequencies of minichromosome loss rates were scored as the frequency of Ade^−^ red colonies. (*f*) Additive defects were observed for the double mutants between *ts-537* and *mis6*, *mis12* or *mis16* (see text).

The CoA synthetic pathway, present in prokaryotes, fungi, plants and animals, consists of five steps, requiring four molecules of nucleotide triphosphate (ATP or CTP) [7–10] (figure 1*a*). Pantothenate is a specific precursor for CoA that is phosphorylated by pantothenate kinase (PANK) to 4′-phosphopantothenate as the initial step. In the next step, phosphopantothenoylcysteine synthetase (PPCS) catalyses the condensation reaction of 4′-phosphopantothenate and cysteine [11]. The third reaction involves decarboxylation reaction to 4′-phosphopantetheine [12]. Finally, the AMP moiety is added to form dephospho-CoA, which is subsequently phosphorylated on the 3′-OH of the ribose to yield CoA [13].

In this study, we report the isolation and characterization of novel temperature-sensitive (ts) mutant strains of fission yeast with defects in mitosis and chromosome segregation, which turned out to contain substitution mutations in the *PPCS* gene (encoded by SPCC4B3.18 and hereafter designated *ppc1*^+^). *Schizosaccharomyces pombe* has proved to be an excellent model system to study the underlying mechanisms of cell division and cycle control, mitosis, meiosis, heterochromatin formation and cellular quiescence, by using powerful genetic approaches [14–22]. Metabolic control can be investigated by metabolomic analysis using mass spectrometry [23–25]. In this study, we measured the level of CoA and provided direct evidence for a considerable decrease in CoA level in mutant cells. Pleiotropic phenotypes observed at molecular and cellular levels are interpreted based on metabolomic results. Our results show that the biosynthetic enzyme for CoA is a super-housekeeping enzyme [26], essential for both proliferation and cellular quiescence. The incidence of breaks in mutant chromosome DNA suggests the requirement of CoA for genome stability and centromere/kinetochore-mediated mitotic progression. Furthermore, as expected from its involvement in the fatty acid biosynthesis and energy metabolism, CoA plays a role in proper maintenance of lipid droplets, the organelles for lipid storage.

## 3. Results

### 3.1. Nuclear division defects observed in *ts-537* mutant cells

One thousand and fifteen ts strains of *S. pombe* were previously isolated, their phenotypes characterized, and some of the genes essential for mitosis, cell growth, cellular quiescence maintenance, glucose metabolism and gene silencing were determined through phenotypic characterizations followed by gene identification and gene product analyses [26–30]. Around 10 per cent of the mutant strain *ts-537* cells, compared with less than 1 per cent of the wild-type (WT), showed the phenotypes of mitotic progression and chromosome segregation defects at the restrictive temperature (36°C), as shown below. Auxotrophy and DNA double-strand break (DSB) damage sensitivity were found at the permissive temperature (26°C). Owing to the unique combination of these chromosomal and nutrient-related phenotypes among so-far isolated mitotic mutants of this organism, we decided to investigate the mutant in this study by using the metabolome approach combined with cellular and molecular analyses.

Staining with the fluorescent 4′,6-diamidino-2-phenylindole (DAPI) revealed the nuclear DNA in fluorescence micrographs of *S. pombe* WT and mutant *ts-537* cells (figure 1*b*). The mutant cells showed an undivided nucleus in one of the septated cytoplasmic spaces (indicated by arrowheads), which were presumably due to temporal cell cycle arrest at mitotic metaphase followed by the displacement of the undivided nucleus and septum formation. Such a phenotype is rarely observed in WT cells [31,32]. Furthermore, an unequal segregation phenotype displaying large and small daughter nuclei (indicated by arrows) was observed, similar to that previously reported in mutants with functional defects in mitotic centromere/kinetochores [27].

### 3.2. Timing of lethal mitotic phenotypes produced after shift to 36°C

For time-dependent determination of defective phenotype appearance, WT and mutant cells were grown at 26°C and then shifted to 36°C. As shown in figure 1*c*(i), the assay for the cell number indicated that the majority of mutant cells (red line) ceased division, whereas the WT cell (blue line) continued to increase. The cell viability of mutant cells (red line, figure 1*c*(ii)) decreased to 20 per cent after 8 h. The highest frequency of the mitotic defects with the displaced nucleus was reached at around 4 h, whereas mitotic defects in the WT were negligible (figure 1*c*(iii)). The appearance of the large and small daughter nuclear phenotype in mutant cells appeared delayed when compared with that of the displaced nucleus, consistent with the fact that the centromeric missegregation phenotype is often observed in mitosis once mutant cells pass the G1-S phase at the restrictive temperature [27].

### 3.3. Segregation defects of sister centromeres

For monitoring the segregation patterns of mitotic sister centromeres, DNA was visualized by green fluorescent protein (GFP) bound to the peri-centromeric regions in the genetically engineered *S. pombe* genome [33]. The GFP-tagged Lac repressor protein was expressed in WT and in the *ts-537* mutant*.* In these strains, repeats of the Lac operator DNA sequence that binds to the repressor had been chromosomally integrated at the peri-centromere of chromosome I. While the two sister centromere signals were always separated in the daughter nuclei in the WT cells (figure 1*d*(i)), the centromere signals were asymmetrically segregated, displaying occasional (approx. 10%) absence in one of the two daughter nuclei in *ts-537* mutant cells after incubation at 36°C (figure 1*d*(ii)).

### 3.4. Loss of minichromosome Ch10-CN2 at 26°C

To quantify the loss rate of an artificial linear minichromosome Ch10-CN2 [34–36] in *ts-537* at 26°C, we used a colony colour assay as shown in figure 1*e*(i). Ch10-CN2, containing the centromere DNA of chromosome III, is stably maintained as an extra chromosome in WT. The colony colour marker *ade6-704* was introduced in the mutant and WT strains harbouring Ch10-CN2. When the minichromosome, carrying the *ade6-704* phenotype*-*suppressing *sup3-5* gene, was lost, the resulting colonies became Ade^−^ and turned red. The frequency of the red-colour colonies, unable to retain Ch10-CN2, was higher in *ts-537* mutant cells even at 26°C. These measurements indicated that the loss rate of Ch10-CN2 (74.3 × 10^−4^ loss events per cell division; figure 1*e*(iii), red column) was markedly (approx. 25-fold) higher compared with the WT (blue column, 3 × 10^−4^).

### 3.5. Additive defects of *ts-537* with centromere/kinetochore mutants

Consistent with the centromere missegregation phenotypes observed in *ts-537*, the mutant genetically interacted with three centromere/kinetochore mutants *mis6*, *mis12* and *mis16* [27,37–39], as shown in figure 1*f*. The spot colony assay indicated that the double mutants *ts-537 mis6-302*, *ts-537 mis12-537* and t*s-537 mis16-53* showed additive defects at 26°C, 30°C and 33°C (the permissive and the semi-permissive temperatures, respectively).

### 3.6. Restoration of cell viability is lost in *ts-537* after the G0 quiescent phase induced by nitrogen starvation

**Figure 2.**
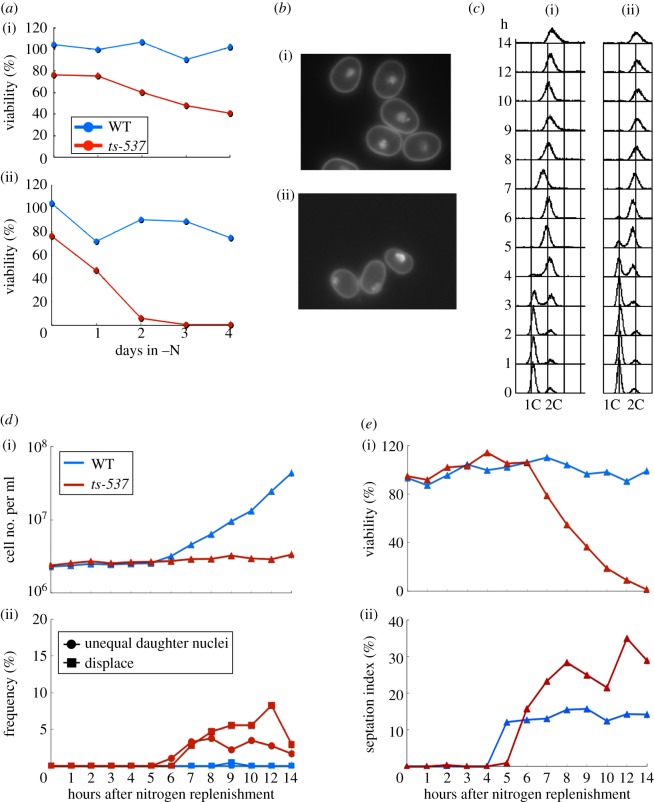
Viability decrease of *ts-537* in the G0 phase and in mitosis. (*a*) Wild-type (WT) and *ts-537* cells were brought into the G0 quiescent state at 26°C (i) under the nitrogen source deficiency for 24 h [26]. Resulting G0 quiescent cultures were shifted to (ii) 36°C for 4 days. As the control, portions of the cultures were kept at 26°C. The cell viability percentages at different time points (days) were obtained by plating cells on the complete medium at 26°C, and calculated as the percentage of the number of formed colonies against the number of plated cells. *ts-537* mutant cells lost the viability within 2 days at the restrictive temperature. (*b*) Light micrographs of non-dividing (i) WT and (ii) *ts-537* G0 cells. Cells were cultured in the nitrogen-deficient medium (EMM2-N) at 26°C for 24 h and transferred to 36°C for 3 days. Cells were then fixed and stained with DAPI. (*c*)(i) WT and (ii) *ts-537* cells, which had been kept in the G0 quiescent medium (EMM2-N) for 24 h at 26°C, were nitrogen source replenished and cultured at 36°C for 14 h. Cells were collected at the time points indicated, and their DNA contents were measured by the Beckton–Dickinson FACscan. See text. (*d*)(i) The cell numbers (blue, WT; red, *ts-537*). (ii) The frequencies of aberrant mitotic cells. Red, *ts-537*; blue, WT. See text. (*e*)(i) Cell viability percentages of WT (blue) and *ts-537* (red). (ii) Septation index of WT (blue) and *ts-537* (red).

To determine whether *ts-537* could maintain the viability in the quiescent G0 phase, we monitored the time course of cell viability under nitrogen starvation [26]. WT and mutant *ts-537* cells first grown in the synthetic Edinburgh minimal medium (EMM2) were transferred to the nitrogen-deficient EMM2-N at 26°C for 24 h, and the resulting quiescent cells, arrested in the pre-replicative G0 phase, were cultivated at either 26°C or 36°C for 4 days. Aliquots of the cultures were taken at intervals, and their cell viability percentage was assayed in the nutrient medium at 26°C (figure 2*a*). The cell viability of *ts-537* was diminished at 36°C after 2 days, whereas the viability of the WT was high even after 4 days, suggesting that *ts-537* failed to restore the cell cycle following a period of G0 maintenance. DAPI-stained cells of WT and *ts-537* are shown in figure 2*b*. No significant difference of cell shape between WT and mutant cells was observed. The nucleus, however, was positioned closely to the plasma membrane in approximately 50 per cent of the mutant cells.

### 3.7. Absence of cell division following G0 exit in replenished *ts-537* cells at 36°C despite S-phase progression

When addressing whether DNA synthesis was affected in *ts-537* mutant cells, WT and *ts-537* strains were brought into a pre-replicative G0 phase by incubation in nitrogen-deficient medium at 26°C (the permissive temperature) for 24 h, as described earlier. The resulting G0 cells were replenished with the nitrogen source in the complete medium (YPD) and cultured at the restrictive temperature (36°C). As shown in figure 2*c*, S phase (DNA replication period) occurred in WT around 4 h and in *ts-537* 5 h after the nitrogen replenishment, suggesting a delay in the onset of S phase.

The cell number of WT started increasing around 8 h, but that of *ts-537* did not (figure 2*d*(i)). Mutant cells producing the earlier-mentioned aberrant mitotic phenotypes were observed after 7–14 h (figure 1*b*(ii)). Despite high cell viability of the WT, *ts-537* viability decreased after 7 h at 36°C (figure 2*e*(i)), suggesting that aberrant mitosis in mutant cells might be a lethal event. The septation index of the *ts-537* mutant started to increase around 6–7 h, about 1 h later than in the WT (figure 2*e*(ii)). Taken together, in *ts-537* at 36°C, the cell viability remained high during DNA synthesis, but decreased in the course of mitosis and septum formation. Cell division/cytokinesis was blocked, suggesting an inhibition of cytokinesis onset owing to aberrant mitosis.

### 3.8. *ts-537* is the mutant of phosphopantothenoylcysteine synthetase

**Figure 3.**
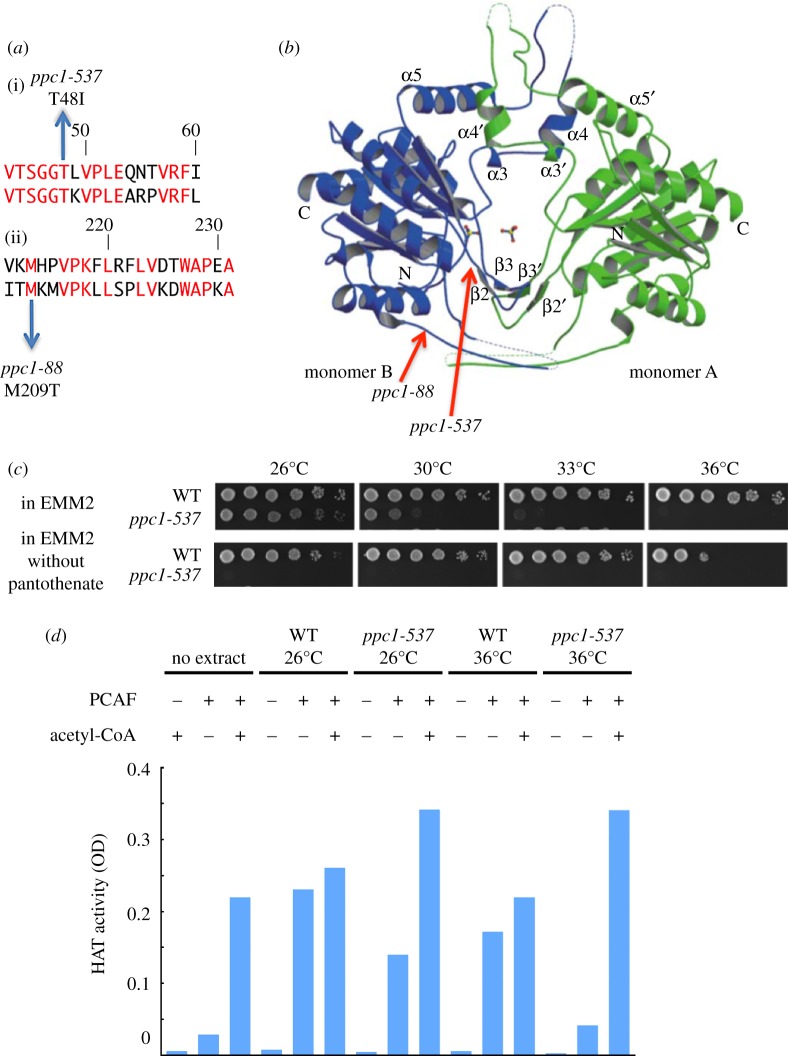
Fission yeast *ts-537* and *ts-88* are mutants of PPCS/Ppc1, auxotrophic to pantothenate, and lack functional acetyl-CoA. (*a*) The substitution mutation sites (T48I and M209T) determined for *ts-537* and *ts-88* strains, respectively, reside within the coding region of PPCS/Ppc1 (top and bottom aligned sequences are *S. pombe* and human, respectively). Identical residues are in red. (*b*) The location of mutation sites in the three-dimensional structure of human PPCS [40]. T48I mutation site in *ppc1-537* locates near the catalytic centre, while the site for *ppc1-88* locates at the periphery of PPCS. (*c*) Pantothenate is needed for *ppc1-537* but not for the WT. Spot tests were done for the WT and *ppc1-537* in the presence or the absence of pantothenate. The exponentially growing cells were spotted by a serial cell concentration on the EMM2 synthetic medium plates either containing or lacking 1 mg l^−1^ pantothenate, followed by incubation at the indicated temperatures for 4 days. (*d*) Cell extracts of growing WT and *ppc1-537* mutant cells were made from the complete culture at 26°C or 36°C. Histone acetyltransferase (HAT) activities of these extracts were measured using the kit containing histone H4 peptide in the addition (+) or the non-addition (−) of human acetyltransferase PCAF recombinant protein and acetyl-CoA. The degree of acetylation that occurred at histone H4 peptide was assayed by the optical density using anti-acetyllysine first antibody and HRP conjugated second antibody. The WT extracts did not need the addition of acetyl-CoA, but the mutant extracts made at 36°C required acetyl-CoA for the HAT activity.

Plasmids that fully rescued the ts phenotype in *ts-537* were isolated using *S. pombe* genomic DNA sequence-containing plasmid library as described previously [27]. The subcloned DNA sequences capable of promoting *ts-537* colony formation at 36°C contained the single gene SPCC4B3.18 that was designated *ppc1^+^* as it encodes PPCS, an intermediate enzyme in the biosynthetic pathway to produce CoA from pantothenate. Genetic analysis by tetrad dissection confirmed that *ts-537* was linked (6.2 cM) to the *sti1^+^* locus that is situated 120 kb apart from the Ppc1/SPCC4B3.18 locus. We then isolated the *ppc1^+^* gene from the genomic DNA of the *ts-537* mutant and its DNA sequence was determined. Only a single nucleotide change was found in the mutant *ppc1* gene that corresponded to the amino acid substitution T48I in the amino-terminal region. This site is highly conserved among human, fly and budding yeast (figure 3*a*(i)). We hence concluded that *ts-537* is a mutant of the PPCS/*ppc1^+^*/SPCC4B3.18 gene and designated it *ppc1-537*.

In addition, the other mutant *ts-88* was also isolated based on its suppression by the *ppc1*^+^ gene-carrying plasmid. The mutant showed a mitotic phenotype similar to that of *ppc1-537*, as shown in electronic supplementary material, figure S1. Subcloned plasmid carrying the *ppc1^+^* gene suppressed the ts phenotype of *ts-88*. The single nucleotide alteration found corresponded to the M209T substitution near the carboxy terminal region (figure 3*a*(ii)). The hydrophobic nature of M209 is conserved in the corresponding genes of other organisms. The phenotype of *ts-88* (hereafter designated *ppc1-88*) was less severe than that of *ppc1-537*, so it was not a target for in-depth investigation.

*Schizosaccharomyces pombe* PPCS/Ppc1 contains 316 amino acids. It is similar to human PPCS (amino acid identity 42%) and budding yeast Cab2 (identity 45%). Two mutation sites in the three-dimensional structure of PPCS/Ppc1 based on the human PPCS [40] are shown in figure 3*b*. The mutation site T48I in *ppc1-537* resides closely to the central catalytic domain, whereas the M209T site in *ppc1-88* is located at the periphery of molecule.

### 3.9. Pantothenate auxotrophy of *ppc1-537* at 26°C

The synthetic medium EMM2 contains pantothenate (1 mg l^−1^). WT *S. pombe* can form colonies regardless of the presence of pantothenate at 26°C. However, colony formation was somewhat retarded at 36°C in the absence of pantothenate (figure 3*c*). By contrast, *ppc1-537* failed to produce colonies at 26°C and 36°C in the absence of pantothenate, whereas the ts phenotype arose in the presence of pantothenate. Therefore, *ppc1-537* turns auxotrophic for pantothenate even at 26°C. The mutant enzyme thus seemed to require a higher concentration of pantothenate to produce sufficient amounts of CoA. A *liz1* mutant that is defective in the *S. pombe* pantothenate uptake was previously shown to require the addition of pantothenate [41]. The phenotypes of *liz1* were slow growth, mitotic defects in the presence of hydroxyl urea, and delayed cytokinesis.

### 3.10. In *ppc1-537* extracts acetyl-CoA usable for the histone acetyltransferase assay is scarce

Acetyl-CoA acts as an acetyl group donor for protein acetylation in metabolism. To address whether the level of acetyl-CoA that can be used for the acetyltransferase reaction is low in the *ppc1-537* mutant, a histone acetyltransferase (HAT) enzyme assay was conducted, using *S. pombe* cell extracts as the supplier for acetyl-CoA. WT and *ppc1-537* extracts were incubated with histone H4 peptide and recombinant mammalian P300/CBP-associated factor (PCAF), which possesses acetyltransferase activity [42]. Acetylated H4 peptide was detected by anti-acetyllysine antibody. As shown in figure 3*d*, HAT activity was obtained without acetyl-CoA addition to the WT cell extracts from cultures incubated at 26°C and 36°C, whereas *ppc1-537* was cultured only in 26°C for extraction. The PCAF HAT activity was decreased fourfold for *ppc1-537* at 36°C and was restored by the addition of pure acetyl-CoA in the HAT assay. The level of acetyl-CoA usable for the histone acetylation assay was thus deficient in the mutant extracts prepared from cells cultured at 36°C.

### 3.11. The accumulation of 4′-phosphopantothenate in *ppc1-537*

**Figure 4.**
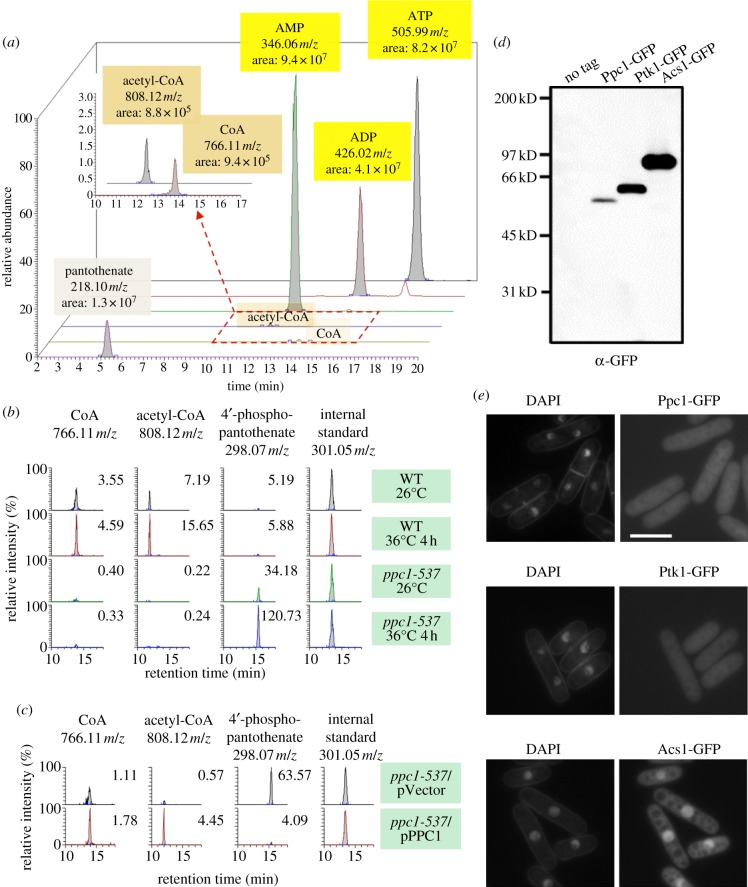
Detection of CoA, acetyl-CoA and Ppc1/PPCS in *Schizosaccharomyces pombe*. (*a*) Metabolomic analysis was performed for the specimens for LC/MS prepared as described [23,24] (§5) using an LTQ Orbitrap mass spectrometer. The amounts of ATP, ADP, AMP, CoA, acetyl-CoA and pantothenate are shown with the *m*/*z* values, the integrated peak areas and the retention times (min). (*b*) WT and *ppc1-537* grown at 26°C were transferred to 36°C for 4 h in the EMM2 medium. Metabolites were extracted from WT and mutant cells and analysed. The levels of CoA, acetyl-CoA and 4′-phosphopantothenate are shown, normalized by the amount of internal standard (PIPES) using the MZmine 2 software [43]. (*c*) The precursor compound 4′-phosphopantothenate, accumulated in *ppc1-537*, disappeared, and CoA and acetyl-CoA were restored when plasmid expressing the WT *ppc1^+^* gene was introduced into the mutant cells. (*d*) The level of Ppc1 protein was measured using the chromosomally integrated strain with the Ppc1 tagged with GFP and expressed under the native promoter. Two other integrant strains carrying Ptk1-GFP or Acs1-GFP are shown as the control. (*e*) Localization of Ppc1-GFP chromosomally integrated and expressed under the native promoter. Cells were cultured at 26°C and the GFP signal was observed with DAPI co-staining. Scale bar, 10 μm.

To characterize mutant phenotypes, the metabolomic analysis recently developed for *S. pombe* [23–25] was applied to assay the level of CoA and acetyl-CoA present in WT and mutant cell extracts, using an LTQ Orbitrap mass spectrometer. In *S. pombe*, using our extraction and detection conditions, the levels of CoA and acetyl-CoA were relatively low, nearly 1/100-fold in the peak areas in comparison with other abundant metabolites such as ATP, ADP and AMP (figure 4*a*). However, we could detect reproducible peaks of ionized CoA, acetyl-CoA and their precursor compounds in repeated experiments [24]. The identity of CoA and acetyl-CoA peaks was verified using purchased standard compounds.

WT and *ppc1-537* mutant cells were grown at 26°C, then transferred to 36°C and collected after 4 h. In the mutant cell extracts, the levels of CoA and acetyl-CoA detected were low (figure 4*b*; the numbers indicate normalized peak areas). By contrast, the level of 4′-phosphopantothenate, the substrate of Ppc1 enzyme in the pathway, was detected at 26°C and sharply increased at 36°C. However, 4′-phosphopantothenate was barely detected in the WT at both 26 and 36°C, indicating that 4′-phosphopantothenate might be rapidly metabolized as an intermediate for the synthesis of CoA in WT cells.

Next, we examined whether 4′-phosphopantothenate level decreased following the introduction of WT *ppc1*^+^ gene under the native promoter into mutant cells using plasmids. As shown in figure 4*c*, the level of 4′-phosphopantothenate became negligible upon *ppc1*^+^ gene introduction into *ppc1-537* cells followed by incubation at 26°C. Taken together, Ppc1 appeared to act at the predicted step of the CoA synthetic process. If Ppc1 was defective, the precursor compound 4′-phosphopantothenate was highly accumulated as a consequence of blockage of the responsible enzymatic step.

### 3.12. Detection of Ppc1 protein and its whole cellular localization

To identify the Ppc1 protein in *S. pombe* cells, the *ppc1*^+^ gene was tagged at the carboxy terminus with GFP by chromosomal integration and expressed under the native promoter. For comparison, the genes for SPBC4B4.01c/Ptk1 (designated Ptk1, PANK) and SPCC191.02c (designated Acs1, acetyl-CoA synthetase that forms acetyl-CoA from acetate and CoA) were GFP-tagged and chromosomally integrated under the native promoter. Figure 4*d* depicts these three proteins detected by immunoblot using an antibody against GFP in the extracts of growing WT *S. pombe* cells. Single bands were detected at the expected MWs. Ptk1/SPBC4B4.01c and particularly Acs1/SPCC191.02c were much more abundant than Ppc1.

The intracellular localization of Ppc1, Ptk1 and Acs1 was determined using these integrated GFP-tagged strains expressed under the native promoter. The GFP signals of both Ppc1 and Ptk1 were observed in the whole cell (figure 4*e*), whereas Acs1 was enriched in the nuclear chromatin. These localization results are similar to those reported [44].

### 3.13. Hypersensitivity to double-strand break-causing phleomycin and bleomycin

**Figure 5.**
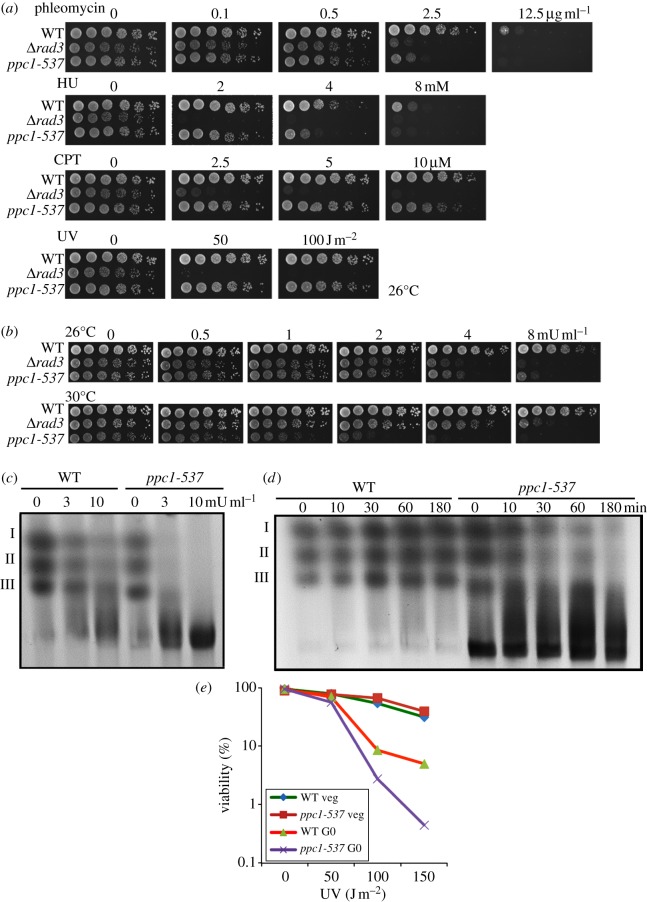
Hypersensitivity of *ppc1-537* to bleomycin that causes double-strand break (DSB). (*a*) The WT, *ppc1-537* and *Δrad3* mutant cells were grown at 26°C; spotted on the rich YPD medium containing DNA-damaging agents, phleomycin, hydroxyurea (HU) or camptothecin (CPT); irradiated by UV rays; and cultured at 26°C for 4 days. (*b*) Effect of bleomycin on *ppc1-537* and *Δrad3* was tested. Cells were grown exponentially at 26°C and then spotted at serial cell concentration on the YPD plates containing bleomycin (the concentration range, 0–8 mU ml^−1^), followed by incubation at 26°C or 30°C for 4 days. (*c*) Pulsed field gel electrophoresis (PFGE) of *ppc1-537* mutant chromosome DNAs. PFGE analysis of *S. pombe* chromosomes was carried out. WT and *ppc1-537* mutant cells were cultured at 26°C for 3 h after the addition of 0–10 mU ml^−1^ bleomycin to the medium. *Schizosaccharomyces pombe* has three chromosome DNAs that were degraded in the medium containing 3 or 10 mU ml^−1^ bleomycin. (*d*) Time course analysis of chromosome breakage by bleomycin was monitored by PFGE analysis. WT and *ppc1-537* mutant cells were cultured at 26°C. Cells were harvested at indicated time points after addition of 3 mU ml^−1^ bleomycin. (*e*) The G0 cells of *ppc1-537* were more sensitive to UV than the WT. See text.

We found that *ppc1-537* was hypersensitive to certain DNA-damaging agents at the permissive temperature. As shown in figure 5*a*, *ppc1-537* was sensitive to 2.5 μg ml^−1^ DNA-breaking phleomycin at 26°C. It was moderately sensitive to 4 mM hydroxyurea (DNA replication inhibitor), but hardly sensitive to 5 µM camptothecin and to 50–100 J m^−2^ ultraviolet (UV) ray. The *rad3* deletion (Rad3 is an ATR-like checkpoint kinase [45]) is the control strain for DNA damage sensitivity.

It was also found that *ppc1-537* was sensitive to bleomycin that cleaves double-strand DNA. As shown in figure 5*b*, *ppc1-537* was sensitive to 4 mU ml^−1^ bleomycin at 26°C. The sensitivity was comparable to that of *Δrad3*. At 30°C (semi-permissive temperature), *ppc1-537* could produce colonies in the absence of the drug, but failed to produce colonies in the presence of 2 mU ml^−1^ bleomycin, showing that *ppc1* mutant was more sensitive than *Δrad3* to bleomycin at this temperature. The electronic supplementary material, figure S2 shows that the sensitivity of *ppc1-537* to bleomycin at 26°C was restored by plasmid pPPC1 carrying the *ppc1*^+^ gene, but not by vector plasmid.

To monitor the occurrence of DSB *in vivo* for chromosomal DNA when the DSB-causing drug was added to the culture medium, pulsed field gel electrophoresis (PFGE) was used (§5). In the presence of 3 mU ml^−1^ bleomycin at 26°C for 3 h, the intensity of the three intact chromosome bands (I, II, III) greatly decreased in *ppc1-537* (figure 5*c*, right; the WT control pattern is shown at left).

The time course disappearance of the three chromosomes I, II and III was examined at 26°C in mutant cells in the presence of 3 mU ml^−1^ bleomycin at 26°C (figure 5*d*). In the WT control, the three bands were still intact at 180 min. In *ppc1-537* mutant, however, broken DNA appeared already after 10 min. On the other hand, most chromosomal DNA had been broken about 60 min following 3 mU ml^−1^ bleomycin addition at 26°C.

### 3.14. *ppc1-537* is UV-sensitive in G0 quiescent cells

While *ppc1-537* was insensitive to UV irradiation in the growth culture medium, we found that the mutant cells became sensitive to 100 J m^−2^ UV while residing in the quiescent G0 phase, as shown in figure 5*e*. The *ppc1-537* mutant cells were cultured for 24 h at 26°C in the absence of a nitrogen source. Cells were then irradiated by UV (0–150 J m^−2^) at 26°C. The difference of sensitivity between the WT and *ppc1-537* was about tenfold in the G0 phase, while the sensitivity in the WT did not differ significantly from mutant cells during vegetative phase. The nitrogen-starvation-induced G0 phase entry of *S. pombe* was previously shown to be hypersensitive to DNA-damaging agents as the damage repair through homologous recombination was largely missing due to the absence of post-replicative DNA [46].

### 3.15. Greatly diminished histone H3 and H4 acetylation in *ppc1-537* cells

Because both levels of CoA and also acetyl-CoA were greatly diminished in *ppc1-537* mutant cells even under permissive temperature, in the next step we examined whether histone acetylation decreased in mutant cells. Immunoblot analysis was done using antibodies against acetylated histones. Cell extracts were prepared from three strains: WT, histone deacetylase mutant *clr6-1* [47] and *ppc1-537*. The cells were shifted from 26°C to 36°C for 0–12 h, and three specific antibodies against acetylated histone H3 or H4 (AcH4, AcH3K9, AcH3K14) were used to detect histone acetylation. For the loading control, antibodies against the carboxy terminus of histone H3 (H3) and Cdc2 (PSTAIRE) were used (§5).

**Figure 6.**
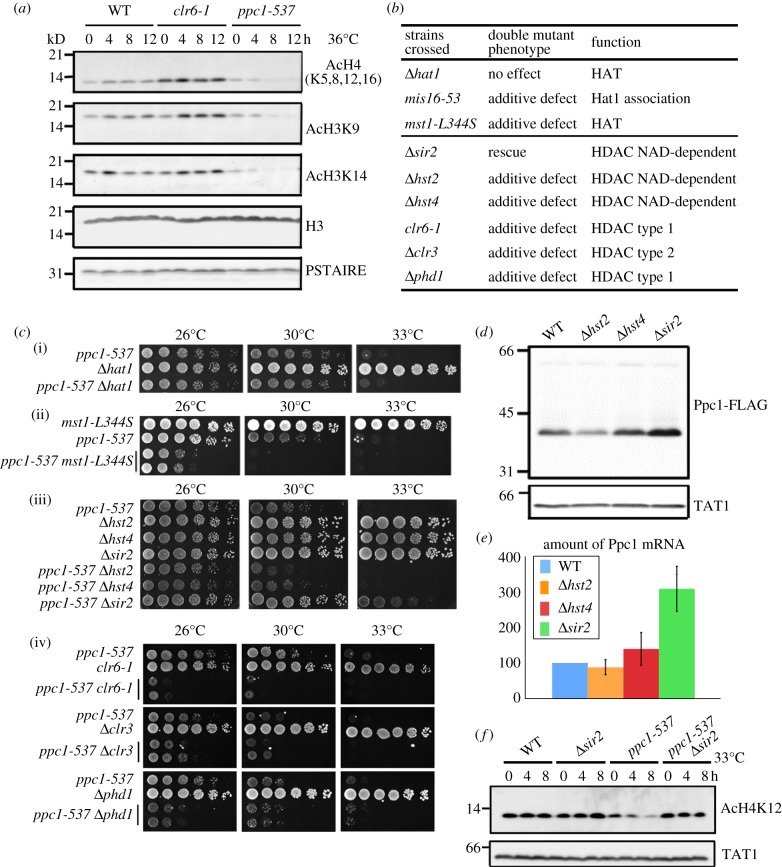
Histone acetylation is diminished in *ppc1-537* that strongly interacts with histone acetyltransferase and deacetylase mutants. (*a*) WT, *clr6-1* and *ppc1-537* mutant strains were first grown at 26°C, then transferred to 36°C for 0, 4, 8 and 12 h, and harvested. Their extracts were prepared and immunoblotted using antibodies against acetylated histone H3 and H4 as indicated and antibodies against histone H3. Cdc2 antibody PSTAIRE was the loading control. (*b*) Results of the double mutants between *ppc1-537* and acetyltransferase mutants *Δhat1* and *mst1-L344S*, and six deacetylase mutants. Additive defects were observed except for the case of double mutant with *Δsir2*, which was rescued, and with *Δhat1*, which showed no effect. (*c*) The single and double mutants of *ppc1-537* and acetyltransferase (*hat1*, *mst1*) and deacetylase (*hst2*, *hst4*, *sir2*, *clr6*, *clr3*, *phd1*) mutants. Cells grown exponentially were diluted, spotted on YPD plate (two spots for the double mutant), and then incubated at 26°C, 30°C or 33°C. (*d*) Protein and (*e*) mRNA transcript levels of FLAG-tagged Ppc1 in the genetic background of WT, *Δhst2*, *Δhst4* and *Δsir2*. The level of Ppc1-FLAG chromosomally integrated and expressed under the native promoter was determined by anti-FLAG antibody. (*f*) The levels of acetylated histone H4 K12 in WT, *Δsir2*, *ppc1-537* and the double mutant were determined by antibody against acetylated H4K12. Anti-tubulin antibody TAT1 was determined as the loading control.

In *clr6-1* mutant cells, the level of histone H3 and H4 acetylation moderately increased (figure 6*a*), as reported [48]. By contrast, the levels of histone acetylation detected by antibodies against acetylated histones H4 AcH4 (at K5, K8, K12 and K16), AcH3 (K9) and AcH3 (K14) [49] were all diminished in *ppc1-537* for 0–12 h at 36°C, while the histone H3 protein level did not change. These results were consistent with a notion that histone acetylation was diminished in *ppc1-537* owing to the deficiency of CoA and acetyl-CoA.

### 3.16. Genetic interactions of *ppc1-537* with histone acetyltransferase and deacetylase mutants

To obtain information about genetic interactions with HAT, double mutants containing two mutations of HAT *mst1-L344S* [50] and *Δhat1* [51] were constructed. Mst1, a homologue of budding yeast Esa1 [52], is required for damage response and chromosome segregation. Hat1, a homologue of budding yeast Hat1, is required for histone H4 acetylation. Mis16 is an essential histone H4 chaperone, and its ts mutant is defective in chromosome segregation and centromeric histone H3 (CENP-A) loading to the centromere [27]. As shown in figure 6*b*,*c*(i,ii), *ppc1-537* caused synthetic lethality when combined with *mst1-L344S* but not with *Δhat1*. Note that *ppc1-537* was synthetically defective with *mis16* mutant, as described in figure 1*f*.

*Schizosaccharomyces pombe* has six histone deacetylases (HDACs). We constructed double mutants of *ppc1-537* with these and examined their ts phenotypes (figure 6*b*,*c*). The additive defects were found in five double mutants. The severe additive phenotypes were found in the type 1 and type 2 HDACs, *clr6*, *clr3* and *phd1*. However, double mutants with NAD-dependent HDACs *ppc1 hst2* and *ppc1 hst4* only showed a weak additive defect at both 26°C and 30°C. Interestingly, *ppc1-537* combined with *Δsir2* showed a clear rescue of the ts phenotype at 30°C and 33°C. Sir2 belongs to the group of HDACs dependent on NAD for their activity.

As the degree of rescue for the double mutant *ppc1* *Δsir2* was strong, we performed further experiments. It was noticed that the protein and mRNA levels of Ppc1 significantly increased in *Δsir2* deletion mutant. The levels were higher than those of WT cells (figure 6*d*,*e*). The level of acetylated histone H4 K12 was low in *ppc1-537*, but restored in the *ppc1* *Δsir2* double mutant cells at semi-permissive temperature (33°C; figure 6*f*). Histone H4 K12 is not a direct target of fission yeast Sir2. This recovery of histone acetylation might occur through the upregulation of Ppc1 by the deletion of Sir2.

### 3.17. The number of lipid droplets decreased in *ppc1-537*

**Figure 7.**
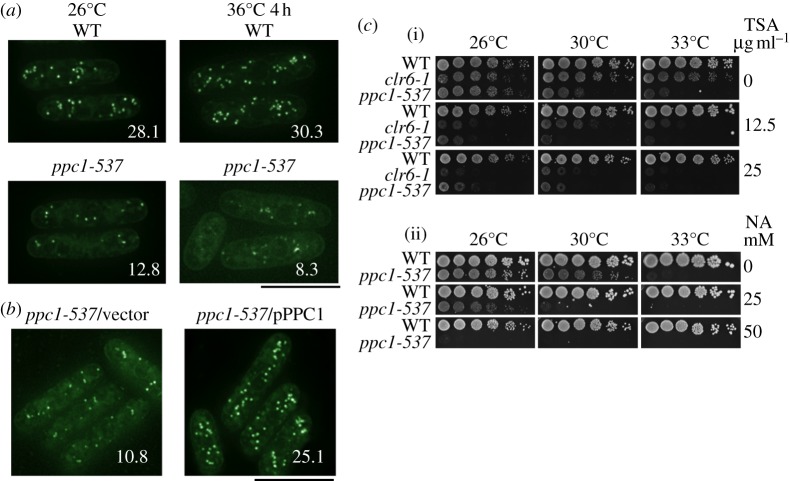
Lipid homeostasis is impaired in *ppc1-537* mutant. (*a*) WT and *ppc1-537* cells grown at 26°C and at 36°C for 4 h were stained by nile red [53] to visualize lipid droplets. The number indicates the relative abundance of lipid droplets in WT and *ppc1-537* cells. Scale bar, 10 μm. (*b*) Recovery of lipid droplets stained by nile red upon the transformation of *ppc1^+^* gene. Scale bar, 10 μm. (*c*)(i) *ppc1-537* mutant was sensitive to TSA at 26°C. The sensitivity was similar to that of *clr6-1* mutant at 26°C. (ii) *ppc1-537* was sensitive to nicotinamide (NA) at 26–30°C, while the WT cells normally grew at the same temperature.

The neutral lipids (triacylglycerol and cholesteryl esters) serve as an energy reserve, and these molecules are stored in lipid droplets. Acetyl-CoA is also required for the production of these metabolites, and we found that lipid droplets stained by nile red [53] showed a striking difference between WT and *ppc1-537* mutants (figure 7*a*). The number of lipid droplets was already relatively low in *ppc1-537* mutant at the permissive temperature. At restrictive temperature, the number further decreased, while the number did not change in WT cells. Introduction of the plasmid carrying the *ppc1*^+^ gene into the mutant cells restored the number of lipid droplets (figure 7*b*). These results suggest that lipid droplet homeostasis requires proper CoA biosynthesis.

### 3.18. *ppc1-537* is sensitive to the histone deacetylase inhibitors trichostatin A and nicotinamide

*Schizosaccharomyces pombe* chromosome segregation is affected by inhibitors acting against HDACs. HDAC inhibitor trichostatin A (TSA) [54] is a potent inhibitor, and promotes normal and abnormal sister chromatid separation by affecting APC/cyclosome and adherin. As shown in figure 7*c*(i), despite normal growth of the WT strain, *ppc1-537* mutant was hypersensitive to TSA (12.5 μg ml^−1^), similar to the *clr6-1* mutant at 26°C. Additionally, mutant cells were sensitive to 25–50 mM nicotinamide, which inhibits NAD-dependent HDACs such as Sir2 [55,56]. Again, WT cells were equally unaffected by this drug concentration, as shown in figure 7*c*(ii). Thus, *ppc1-537* was sensitive to two distinct types of HDAC inhibitors.

## 4. Discussion

The name of pantothenate or pantothenic acid derives from the Greek word *pan* or *pantos*, meaning all or universal. This compound is present in all known organisms as the specific precursor of CoA, a fundamentally essential coenzyme in cellular metabolism. The enzyme PANK produces phosphopantothenate by phosphorylation, and PPCS subsequently forms phosphopantothenoylcysteine by the ligation reaction with cysteine and CTP or ATP. The following three reactions lead to the synthesis of CoA. These reactions are conserved and absolutely essential for life [57–61]. While fungi such as *S. pombe* produce pantothenate from pyruvate and beta-alanine [41], animals do not, taking pantothenate entirely from the food. Therefore, pantothenate is also called vitamin B5. In this study, we isolated two *S. pombe* ts mutant strains (*ppc1-537*, *ppc1-88*) defective in PPCS through gene cloning by transformation and sequencing. In addition, one ts mutant strain (*ptk1-201*) defective in PANK was isolated (T. Nakamura 2010, unpublished result). Mass spectrometry analysis established that the levels of CoA in these mutant cell extracts are greatly diminished, and that the level of phosphopantothenate is decreased in *ptk1-201* owing to the lack of phosphorylation of pantothenate, while it is increased in *ppc1-537* owing to the defect in the subsequent reaction. Our results support the conclusion that the cellular phenotypes of these mutants are due to the loss of the activities of PANK and PPCS, which causes the diminished level of CoA.

After CoA is synthesized, it can be acylated at the thiol group (SH) by various compounds, giving rise to thioester forms. Acetyl, acetoacetyl, succinyl, glutaryl, malonyl, hydroxymethylglutaryl, coumaroyl, crotonyl, propionyl are examples. Differently acylated CoAs are produced in different places within cells for different physiological purposes. Among acylated CoAs, acetyl-CoA is the most important, as it is essential in so many crucial metabolic pathways. Pyruvate dehydrogenase complex catalyses the reaction of CoA with pyruvate, the end product of glycolysis and produces acetyl-CoA. Acetyl-CoA is then required to start the TCA (Krebs) cycle by citrate synthase that catalyses the reaction
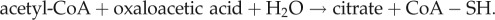
In other words, citrate synthase is a major consumer of acetyl-CoA when TCA cycle is fully active. This reaction occurs in mitochondria.

Acetyl-CoA is also required for the synthesis of fatty acids by fatty acid synthase through its association with an acyl-carrier protein in the very large fatty acid synthase complex. The fatty acid synthesis occurs in cytoplasm. In contrast, fatty acids are oxidized by the process called beta-oxidation and produce acetyl-CoA in mitochondria. Acetyl-CoA is required for the synthesis of cholesterol. Furthermore, acetyl-CoA is required for acetylation of numerous proteins having various functions. As these represent only a portion of whole metabolic events that require acetyl-CoA and other acyl-CoAs, pleiotropic defective phenotype of the mutants deficient in the CoA synthesis may well be expected. Hence, the phenotypes of the reduction of lipid droplets and the defects in cell division cycle and cellular quiescence may be explained owing to the diminished TCA and sugar catabolic processes, and/or fatty acid synthesis. CoA is also required for the super-housekeeping metabolism required for two distinct cell states under division and arrest [26].

In spite of many metabolic roles, the WT level of CoA (and also acetyl-CoA) determined by liquid chromatography–mass spectrometry (LC–MS) is surprisingly low in comparison with other principal metabolites such as ATP or NAD^+^ (NADH). We examined a possibility that CoA and acetyl-CoA might be unstable and largely lost during our preparative procedures of metabolites. When authentic CoA and acetyl-CoA were exogenously added to the samples prior to extractions from cells, the levels sharply increased according to the amounts added: there was no loss of the externally added CoA during the preparation. The pool size of CoA and acetyl-CoA thus appeared to be small: CoA and acetyl-CoA produced *in vivo* might be immediately consumed or changed to other forms. Alternatively, they were tightly bound to proteins or other cellular components within cells, and might be not easily extractable.

The most unexpected findings in the present study are the specific mutant phenotypes that revealed chromosome missegregation in mitosis. The mutant cells did not lose viability during the S phase. The link between the paucity of CoA and the error in chromosome segregation is not immediately grasped with ease. The chromosome segregation defects observed in the CoA biosynthesis mutants are not coincidental to these *S. pombe* mutants, as the isolated mutants show similar segregation defects. It was reported that the fly PPCS mutant caused aberrant mitotic chromosomes and hyper-sensitivity to ionizing radiation [57,58]. Similar mitotic defects were thus observed in the distant organisms. In *S. pombe*, the fatty acid synthesis mutant *cut6* defective in acetyl-CoA carboxylase showed a severe defect in the equal nuclear division [62], indicating that the defect in the fatty acid synthesis metabolism through acetyl-CoA decarboxylation could profoundly affect the mode of spindle dynamics, chromosome segregation and nucleolar division.

The biosynthesis of CoA is closely related to two fundamental aspects of cellular metabolism: the energy production and the sugar/lipid catabolism and synthesis. In this study, we show that the paucity of CoA actually strongly affects the state of chromosomes and also the mode of chromosome segregation in mitosis. While full mechanistic understanding of the relationship between CoA and chromosome must be addressed in a future study, we will discuss several aspects of the relationship revealed in the present study.

We show that *ppc1* mutant is hypersensitive to DNA DSB agents such as bleomycin and phleomycin, suggesting that the mutant chromosome DNA is fragile in the presence of these DSB agents. This may be caused by the decrease in DSB repair efficiency in *ppc1* mutant owing to the hypoacetylation of histones. A previous report showed that hypoacetylation of histones leads to the defect in DNA damage repair. In *Saccharomyces cerevisiae* [63], the HAT Esa1 is required for non-homologous end-joining repair. In higher eukaryotic cells, similar damage repair defects are related to protein acetylation–deacetylation [64,65]. In *S. pombe*, Mst1 similar to Esa1 is related to both DNA damage response and chromosome segregation [50]. We show in this study the synthetic defective phenotype of the double mutant *pcc1 mst1*: Ppc1 may support the DSB repair through the supply of CoA, and concomitantly acetyl-CoA.

Centromeric deposition of CENP-A (centromere-specific histone H3 variant) is known to require appropriate acetylation of histone H3 and/or H4 [66]. ‘Priming’ of the centromere chromatin, a step prior to the deposition of CENP-A, represents histone acetylation. We show that the mutation of Mis16 essential for this step is synthetically lethal with *ppc1* mutant. RbAp46/48, a mammalian orthologue of Mis16, is known to be bound to another histone acetyltransferase Hat1.

The relationship between Ppc1 and HDACs remains to be clarified. The *ppc1* mutant cells are hypersensitive to the decrease in HDACs. First, the *ppc1* mutant was hypersensitive to TSA and nicotinamide, inhibitors of HDACs. Second, five of the six double mutants made between *ppc1* and HDAC mutants constructed in this study revealed additive defects. In contrast, the double mutant *Δsir2 ppc1* rescued the phenotype of *ppc1*. Interestingly, the deletion of Sir2 caused the increase of protein amount and mRNA level of *ppc1^+^*. Sir2 may be the negative regulator for the transcription of *ppc1*^+^ gene. Curiously, Sir2 contains a domain of unknown function called DUF592, which is missing in other HDACs. This DUF592 domain is present in some SIR2 family proteins, and also other functionally unknown proteins found in prokaryotes and eukaryotes. Yet the importance of DUF592 domain in the rescue remains unclear. The relationship between mutants of Ppc1 and other HDACs is not understood. A possible hypothesis is that HDACs producing additive defects with *ppc1* mutation act in parallel with the biosynthesis of acetyl-CoA. Further investigations are needed.

## 5. Material and methods

### 5.1. Strains, media and plasmids

*Schizosaccharomyces pombe* strains used were derived from haploid WT *972* (*h*^−^) and *975* (*h*^+^). The complete YPD, the minimal EMM2 and the sporulation medium SPA were described previously [67]. A *S. pombe* genomic DNA library (a gift of Dr C. Shimoda), containing the budding yeast *LEU2* as the selective marker, was used. For subcloning, a series of plasmids containing each *S. pombe* open reading frame with two FLAG epitopes and one hexahistidine tag was used [44]. Transformation was done using the lithium method [68].

### 5.2. Gene cloning and construction of yeast strains

The genomic DNA library was used to transform mutant strains *ts-88* and *ts-537*, in order to obtain plasmids that suppressed the mutant strains. Plasmids were recovered from resulting transformants. The sequences at the end of the inserts in plasmids defined the inserted genomic sequences. Subcloning established that the SPCC4B3.18 gene was responsible for suppression of *ts-88* and *ts-537*. Genetic linkage analysis was done by tetrad analysis using the marker locus. The result verified the conclusion that the subcloned gene was actually the mutant gene. The amino acid substitutions were confirmed for each mutant gene by sequencing of the mutant genes. The GFP or FLAG tag sequence was inserted at the C terminus of *ppc1^+^ ptk1^+^* and *acs1^+^* genes, and followed by the drug-resistant kanMX6 maker. These DNA fragments were introduced into endogenous loci, and transformants were selected by the resistance to G418. Finally, correct integrations were verified by PCR.

### 5.3. *In vitro* histone acetyltransferase assay

Cell lysates were prepared by glass beads vortexing in the HAT assay buffer (50 mM Tris pH 8.0, 10% glycerol, 0.5 mM EDTA, 1 mM dithiothreitol). After discarding glass beads, lysates were centrifugally filtered through Microcon YM-10 (Millipore). The filtrate, HAT assay kit and recombinant PCAF purchased from Upstate were used for *in vitro* HAT assay, according to the manufacturer's instructions.

### 5.4. Light microscopy

DAPI staining was done as described [69]. To observe cells that expressed the tagged GFP protein, cells were adhered to glass funnel filter and fixed by immersion in 100 per cent methanol at –80°C. After 30 min, phosphate-buffered saline was added to cells for washing cells at 30 per cent methanol dilution. To observe the peri-centromeric DNA, *Lac*I–GFP–NLS was expressed in the presence of thiamine [38] and bound to the *Lac*I binding sequences at the *lys1* locus near cenI [70,71].

### 5.5. Minichromosome stability assay

The stability of minichromosome Ch10-CN2 [34] in fission yeast strain was determined as described [35]. Briefly, cells were cultured in the selective minimal medium at 26°C, then diluted and transferred to non-selective-rich medium and cultured at 26°C. The ratio of Ade^+^ to total cells was obtained after the transfer to the rich medium by plating cells and counting red colonies.

### 5.6. Cell viability assay in the G0 state

Cells were first grown in the minimal medium (EMM2) at 26°C, and then transferred to the nitrogen-deficient EMM2-N medium at 26°C for 24 h. These cultures were split into two and transferred to 26°C and 36°C. Portions of the cultures were taken at each time points and plated on complete YPD medium plates at 26°C. Cell viability was calculated as a percentage of the number of formed colonies against the number of plated cells.

### 5.7. Extraction of CoA and acetyl-CoA for mass spectrometry analysis

*Schizosaccharomyces pombe* cells were grown in the EMM2 medium, and 40 ml (5 × 10^6^ cells ml^−1^) of the culture was used for the metabolite extraction using the method described previously [24], with modifications. Cells were harvested by Omnipore membrane filter (Millipore) and washed with 1 ml Milli-Q water. The filter was plunged into 1.5 ml of cold 50 per cent methanol (−40°C) containing 10 μl of 0.4 mM internal standard piperazine-N,N′-bis(2-ethanesulphonic acid) (PIPES), then frozen in liquid nitrogen followed by thawing on ice. Cells were resuspended by brief vortex and the membrane filter was removed. The suspension was centrifuged at −20°C. The supernatant was collected and 1 ml of cold 50 per cent methanol (−40°C) was added to the pellet and cells were resuspended by brief vortex. The suspension was centrifuged at −20°C, and both supernatants were pooled. Supernatants were centrifugally filtered through an Amicon 5-kDa cut-off filter (Millipore). The filtrate was evaporated and dissolved in 40 μl of 50 per cent acetonitrile.

### 5.8. Liquid chromatography–mass spectrometry analysis

LC–MS data were obtained using a Paradigm MS4 HPLC system (Michrom Bioresources) coupled to an LTQ Orbitrap hybrid ion-trap/Fourier transform mass spectrometer (Thermo Fisher Scientific). LC separation was performed on a ZIC-pHILIC column (Merck SeQuant; 150 × 2.1 mm, 5 µm particle size). Acetonitrile (solvent A) and 10 mM ammonium carbonate, pH 9.3 (solvent B) were used as a mobile phase, with a gradient elution from 20 per cent B to 80 per cent B in 30 min with the 100 µl min^−1^ flow rate. For MS detection, an electrospray ionization source was used and operated in negative ionization mode. Spray voltage was set to 2.5 kV, capillary temperature to 350°C. N_2_ was used as sheath gas. Mass spectrometer was operated in full scan mode with a 100–1000 *m/z* scan range. Raw data were analysed using the MZmine 2 software [43]. Metabolite peak areas were normalized by the peak areas of the internal standard (PIPES). Peaks of CoA and acetyl-CoA were identified by their accurate *m*/*z* values, and identification was verified using the pure standard CoA and acetyl-CoA.

### 5.9. Pulse-field gel electrophoresis

*Schizosaccharomyces pombe* cells (1 × 10^8^) were harvested and chromosome DNA samples were prepared as described [72]. Chromosomal DNA embedded in 1 per cent agarose plug was run on 1 per cent SeaKem Gold Agarose (Lonza) gel in 1× TAE buffer using CHEF Mapper (Bio-Rad). Electrophoresis was carried out at 14°C with 2 V cm^−1^ voltage. Block 1 was done for 8 h with 96° angle and 1200 s switching time. Block 2 was done for 8 h with 100° angle and 1500 s switching time. Block 3 was done for 8 h with 106° angle and 1800 s switching time. The DNA was stained with EtBr.

### 5.10. Detection of histone acetylation

Antibodies specific for acetylated histone (Millipore Upstate 06-866, 07-450, 07-353 and 06-1352) were used to monitor histone acetylation in cell extracts. Antibody specific for histone H3 (GeneTex GTX21791) was used to detect the amount of histone H3.

## 6. Acknowledgements

We are greatly indebted to K. Ekwall and S. Forsburg for *S. pombe* strains and M. Yoshida for plasmids. We thank C. Starzynski for reading the manuscript. The present work was partly supported by the CREST Research Fund from the Japan Science and Technology Corporation when M.Y. was at Kyoto University.

## Supplementary Material

Figure S1

## Supplementary Material

Figure S2
