# Supplementary figures and images for: Impaired coenzyme A synthesis in fission yeast causes defective mitosis, quiescence-exit failure, histone hypoacetylation and fragile DNA
Source: Open Biol. 2012 Sep;2(9):120117. doi: 10.1098/rsob.120117 (PMC3472395; doi:10.1098/rsob.120117)

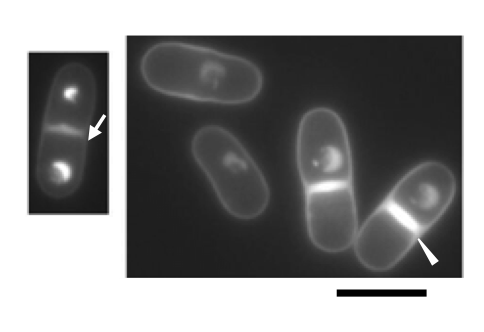

Supplement: Figure S1 [file rsob120117-s1.tif]

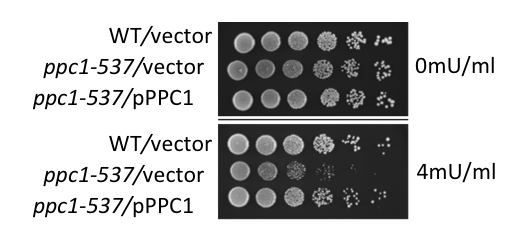

Supplement: Figure S2 [file rsob120117-s2.tif]
